# Biofuels and Their Co-Products as Livestock Feed: Global Economic and Environmental Implications

**DOI:** 10.3390/molecules21030285

**Published:** 2016-02-29

**Authors:** József Popp, Mónika Harangi-Rákos, Zoltán Gabnai, Péter Balogh, Gabriella Antal, Attila Bai

**Affiliations:** 1 Institute of Sectoral Economics and Methodology, Faculty of Economics and Business, University of Debrecen, Debrecen 4032, Hungary; popp.jozsef@econ.unideb.hu (J.P.); rakos.monika@econ.unideb.hu (M.H.-R.); balogh.peter@econ.unideb.hu (P.B.); 2 Institute of Business Economics, Faculty of Economics and Business, University of Debrecen, Debrecen 4032, Hungary; zoltangabnai@gmail.com (Z.G.); bai.attila@econ.unideb.hu (A.B.)

**Keywords:** bioenergy, biofuels, co-products, feed potential, substitution

## Abstract

This review studies biofuel expansion in terms of competition between conventional and advanced biofuels based on bioenergy potential. Production of advanced biofuels is generally more expensive than current biofuels because products are not yet cost competitive. What is overlooked in the discussion about biofuel is the contribution the industry makes to the global animal feed supply and land use for cultivation of feedstocks. The global ethanol industry produces 44 million metric tonnes of high-quality feed, however, the co-products of biodiesel production have a moderate impact on the feed market contributing to just 8–9 million tonnes of protein meal output a year. By economically displacing traditional feed ingredients co-products from biofuel production are an important and valuable component of the biofuels sector and the global feed market. The return of co-products to the feed market has agricultural land use (and GHG emissions) implications as well. The use of co-products generated from grains and oilseeds can reduce net land use by 11% to 40%. The proportion of global cropland used for biofuels is currently some 2% (30–35 million hectares). By adding co-products substituted for grains and oilseeds the land required for cultivation of feedstocks declines to 1.5% of the global crop area.

## 1. Introduction

**Figure 1:**
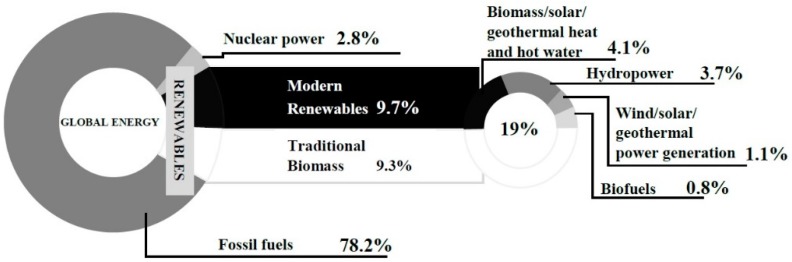
Estimated renewable energy share of global final energy consumption in 2011. Source: [2].

In the last 35 years global energy supplies have nearly doubled but the relative contribution from renewables has increased from 13% to 19%, including about 9.3% from traditional biomass and about 9.7% from modern renewables (Figure 1). The contribution of “modern” renewables (e.g., solar, wind, biofuel) is still a marginal component of total global renewable energy supply, however, they are continuously growing. A major impetus for the development of bioenergy has been the search for alternatives to fossil fuels, particularly those used in transportation [1].

The transport sector is responsible for about 20% of world total energy use. Transport biofuels are currently the fastest growing bioenergy sectors, even though they represent around 3%–4% of total road transport fuel and only 5% of total bioenergy consumption today. Most capacity expansion and financing need is expected for next generation biofuels in the longer term and strong competition from other renewable energy projects with lower risks (wind and solar) can be experienced. Liquid biofuels for transport are generating the most attention, although just a small fraction of biomass is used globally for biofuels production at present [1].

Many countries support production and use of liquid biofuels for transportation to enhance domestic energy security, spur economic development and reduce emissions of greenhouse gases (GHG) and other pollutants. There is an increased focus on advanced biofuels in the past few years. The actual production volumes were not available, the installed production capacity values shows that the global advanced biofuel production capacity increased from 1.58 billion litres in the year 2010 to 4.21 billion litres in the year 2013 [3]. While considerable research and development is under way to commercialize new types of biofuel and feedstocks, the two primary biofuels produced globally today—ethanol and biodiesel—are predominantly derived from agricultural commodities, such as grain, sugar and oilseeds.

**Figure 2:**
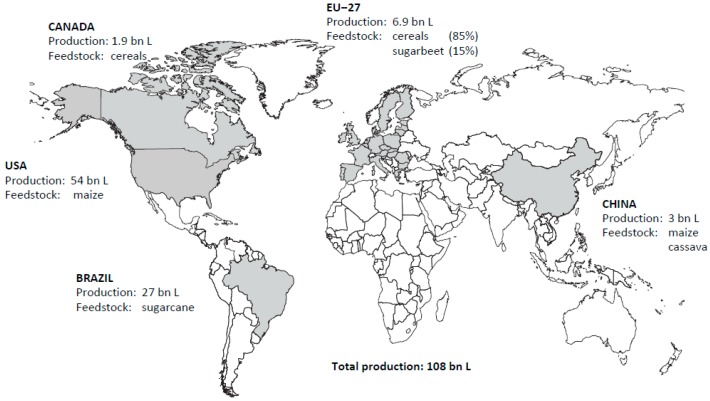
Word fuel ethanol production, average 2012–2014 [5].

**Figure 3:**
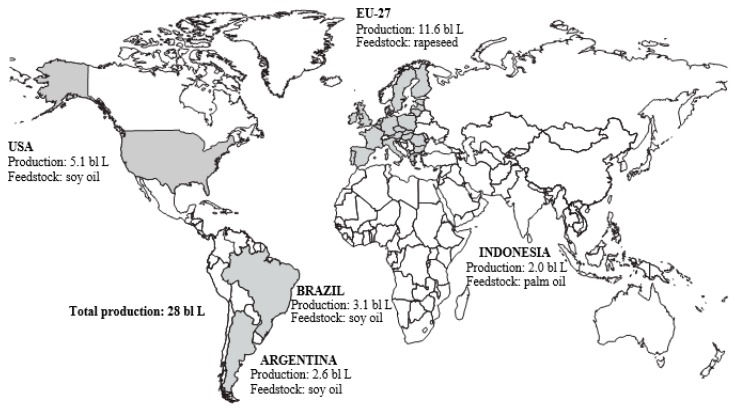
Word biodiesel production, average 2012–2014 [5].

Currently, around 80% of the global production of liquid biofuels is in the form of ethanol. In 2012–2014 on average global fuel ethanol production reached 108 billion litres and global biodiesel production amounted to 28 billion litres (Figure 2 and Figure 3). The two world’s top ethanol producers, the United States and Brazil, accounted for around 75% of total production. Biodiesel production is far less concentrated than ethanol. The European Union remained the centre of global biodiesel production, with 12 billion litres representing 43% of total output. Global expansion of biofuel production is projected to continue during the next decade, although at a slower pace than over the last half decade. Ethanol production in the United States is projected to be relatively flat over the next decade due to the ethanol blend wall and declining gasoline use. Most of the additional ethanol production is expected to take place in Brazil [4]. Indonesia will surpass the United States and Brazil in the latter years of the outlook period to become the second largest biodiesel producer behind the European Union. Global ethanol and biodiesel production are both expected to expand to reach, respectively, almost 135 and 39 billion litres by 2024 [5]. In 2040 the share of biofuels in road transport fuels would range—depending on policies—from 5% to 18% globally, from 11% to 31% in the European Union and from 11% to 29% in the United States [6].

The share of bioethanol in total grains demand—*i.e.*, corn, wheat and other coarse grains—is about 6% of global production. The bulk of the worldwide use of grains in alcohol production comprises maize in the USA and China. However, an increase in the offtake of wheat for fuel ethanol can also be observed in Canada and the European Union. The share of biodiesel in rapeseed, soybean and palm oil demand is around 11% of global vegetable oil production. The share of waste biodiesel feedstocks such as animal fat and used cooking oil increased to 15% in total biodiesel output [7].

Increased biofuel production has led to criticism and concerns about food availability while it is feared that rising demand for agricultural land will lead to deforestation, grassland conversion and increased Greenhouse Gas (GHG) emissions from these land use changes. The main criticism is based on expected impacts of biofuel production following the introduction of dedicated biofuel targets and policies [8,9]. Most models were developed to evaluate agriculture or climate policies and were later adapted to incorporate biofuel production [9,10]. Early applications did not consider generation of co-products (co/by-products of the biofuel production process which are mostly used as animal feed) while second-generation biofuel production technology, at least in early applications, was not included [10,11]. Other restrictions include limited ability to adjust to accelerations in yield improvement or to changes in crop rotation [12]. Central to the debate on the impact of biofuel production is the way increased biomass requirements are to be met by area expansion, yield improvement or by increased cropping intensity. While the exact consequences of these limitations remain unclear economic model impact assessments of biofuel policies should be considered with care.

Following changes in biofuel policies a strong expansion in biofuel production was observed in the USA, the EU, China, and many other countries. These increases, however, were not sufficient to fully satisfy biofuel policy objectives in the USA and the EU. Further expansion of biofuel production is expected in the USA, Brazil, Argentina, and the EU. Co-product generation in early biofuel impact assessments was ignored leading to an overestimation of land requirements and GHG emissions. The output of feed co-products is relatively high in the USA, the EU and China due to the large share of grains used in ethanol production with high feed yields. It is low in Brazil where ethanol production is dominated by sugarcane which generates no feed co-products. Co-product yields are low for rapeseed and soybean used in the biodiesel industry. Estimates on impacts of biofuel production often use models with limited ability to incorporate economic and environmental implications by ignoring generation of co-products from biofuel production. Significant research and development efforts are under way to commercialize “second generation” feedstocks and biofuels, however, these are unlikely to be produced in quantity in the short term. Furthermore, the co-products from many of these new feedstocks are not likely to have applications in the animal feed market [4,13].

The review is organized as follows: first, it describes bioenergy potential including competition between conventional and advanced biofuels. Next, it presents feedstocks and co-products of biofuel production. Furthermore, attributes of co-products are carefully analysed based on economic and environmental implications of biofuel production. This is followed by materials and methods, and some conclusions.

## 2. Results and Discussion

### 2.1. Bioenergy Potential

**Figure 4:**
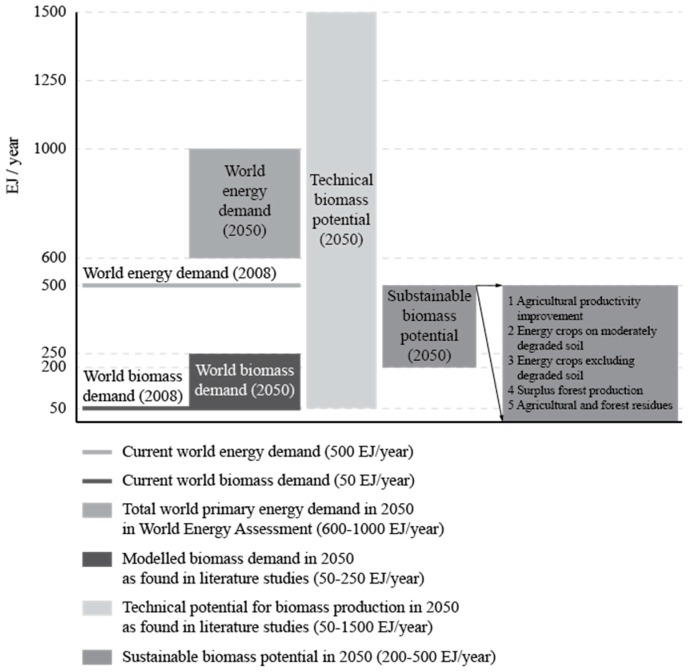
Global bioenergy sources [14].

The worldwide potential of bioenergy is limited because all land is multifunctional and land is also needed for food, feed, timber and fibre production, as well as for nature conservation and climate protection. In addition, the use of biomass as an industrial feedstock (e.g., plastics) will become increasingly important. At present, some 55 EJ/year of bioenergy are produced globally. Modern forms of bioenergy in use in 2011 amounted to 23.6 EJ as heat, biofuel and electricity. An additional 31.4 EJ of traditional biomass was used very inefficiently for cooking/heating in poor rural areas, mainly in Africa [2]. Based on this diverse range of feedstocks, the technical potential for biomass is estimated in the literature to be possibly as high as 1500 EJ/year by 2050 (Table 1). However, most biomass supply scenarios that take into account sustainability constraints, indicate an annual potential of between 200 and 500 EJ/year (excluding aquatic biomass owing to its early state of development), representing 40 to 100% of the current global energy use [14]. Forestry and agricultural residues and other organic wastes (including municipal solid waste) would provide between 50 and 150 EJ/year, while the remainder would come from energy crops, surplus forest growth, and increased agricultural productivity (Figure 4).

Projected world primary energy demand by 2050 is expected to be in the range of 600 to 1000 EJ/year compared to about 500 EJ in 2008. The expert assessment suggests potential deployment levels of bioenergy by 2050 in the range of 100–300 EJ/year. However, there are large uncertainties in this potential, such as market and policy conditions, and there is strong dependence on the rate of improvements in the agricultural sector for food, fodder and fibre production and forest products. The entire current global biomass harvest would be required to achieve a 200 EJ/year deployment level of bioenergy by 2050. Scenarios looking at the penetration of different low carbon energy sources indicate that future demand for bioenergy could be up to 250 EJ/year [21] It is reasonable to assume that biomass could sustainably contribute between a quarter and a third of the future global energy mix (Figure 4). The total annual aboveground net primary production (the net amount of carbon assimilated in a time period by vegetation) on the Earth’s terrestrial surface is estimated to be about 35 Gt carbon, or 1260 EJ/year assuming an average carbon content of 50% and 18 GJ/t average heating value [22], which can be compared to the current world primary energy supply of about 500 EJ/year [14]. All harvested biomass used for food, fodder, fibre and forest products, when expressed in equivalent heat content, equals 219 EJ/year [23]. The global harvest of major crops (cereals, oil crops, sugar crops, roots, tubers and pulses) corresponds to about 60 EJ/year and the global industrial roundwood production corresponds to 15 to 20 EJ/year [24].

Availability of land for non-food crops will be determined by increased yield potential, reducing losses and wastes along the food chain and lower inputs. However, these volumes will remain limited relative to total energy and transport sector fuel demand. Limited biomass resources will be allocated to the sector (materials, chemicals, energy) that is most able to afford them. This will depend on the price of existing fossil fuel products and the relative cost of converting biomass into substitute final fuels such as bio-derived electricity, ethanol blends, biodiesel and bio-derived jet fuel. It will also depend on factors such as cost of alternative fuel and energy sources, government policies including excise rates, and the emission intensity of each sector. The sustainable use of residues and wastes for bioenergy, which do not require any new agricultural land and present limited or zero environmental risks, needs to be encouraged and promoted globally. Several factors may discourage the use of these “lower-risk” resources. Using residues and surplus forest growth, and establishing energy crop plantations on currently unused land, may prove more expensive than creating large-scale energy plantations on arable land. In the case of residues, opportunity costs can occur, and the scattered distribution of residues may render it difficult in some places to recover them [25]. Whatever is actually realised will depend on the cost competitiveness of bioenergy and on future policy frameworks, such as greenhouse gas emission reduction targets. The uptake of biomass depends on biomass production costs, logistics, and resource and environmental issues [18].

### 2.2. Competition between Conventional and Advanced Biofuels

Significant development of advanced biofuels is necessary for diversification and decarbonisation of transport in the longer term. Although a number of projects on 2nd generation bioethanol ended with the opening of pilot and demonstration plants around the world and in spite of several proclamations, none of them is operating at the industrial scale. To make this possible, further reductions in processing costs will be necessary to achieve a product that is competitive with 1st generation bioethanol. By-products, e.g., lignin separated after pre-treatment procedure can be used to generate energy for ethanol plant operations or used as a dispersant and binder in concrete admixtures, as an alternative to phenolic and epoxy resins, or as the principal component in thermoplastic blends, polyurethane foams or surfactants. A combination of 1st and 2nd generation feedstocks (e.g., corn cobs together with stover) can eliminate bottlenecks and lead to product competitiveness [26].

Biofuels from dedicated lignocelluloses energy crops on marginal land is likely to be a cost-efficient contribution. However, extreme territorial and climatic conditions resistant species and varieties are required. Perennial, herbaceous crops—such as *Miscanthus* sp., *A. donax*—may be sustainable because the annual soil cultivation increases the air’s carbon-dioxide level and these plants can mobilize mineral nutrients from the stems and leaves to rhizomes at the end of growing season, reducing the fertilizer needs. These species can rehabilitate the quality of marginal land [27,28,29,30]. Further requirements for energy plants that growth rapidly produce high biomass without irrigation (high water-use efficiency) and resistance to pests and diseases [31]. On the other hand, some potential biomass plants, such as giant reed, have invasive potential widely in riparian areas. Particularly, during the floods along the rivers, fragments of shoot and rhizome of giant reed can disperse and populate quickly and easily new habitats [32,33,34].

Since 2013 advanced biofuels have made good progress, with nine commercial-scale plants commissioned, seven of these in 2014 and 2015. Policies that mandate blending levels and provide capital incentives, along with the development of secure local feedstock supply chains, have been fundamental. New projects may require oil prices around USD 100/bbl or above to be attractive. There is significant potential to reduce the costs but a sustained long-term policy commitment would be needed, which may face risks from the lower oil price environment [35].

IEA [25] refer to the higher water consumption in the production of advanced compared with conventional biofuels, referring specifically to lignocellulosic ethanol. However, the major part of water consumed would be for feedstock cultivation, so that biofuels from wastes and residues should have a reduced water footprint. Local impacts on water quality and availability should nevertheless be monitored [36]. The processing of feedstocks into biofuels via advanced biochemical or thermochemical conversion routes can require relatively high energy inputs. However, other environmental impacts resulting from the processing of biomass through advanced conversion technologies such as water consumption in processing should be investigated and if necessary be addressed by safeguards [35].

Even as several conventional biofuel production facilities closed their doors, several advanced biofuel production facilities came on line in 2014. These included three new biorefineries using cellulosic plant material (predominantly corn stover) in the United States: POET-DSM, DuPont, and Abengoa. In Brazil, three commercial, 2nd generation biofuel projects started operation: GranBio commercial cellulosic ethanol plant, Raizen/Iogens plant, and Solazyme-Bunge plant. The advanced biofuels industry also faced challenges, however. US-based Kior filed for bankruptcy and decommissioned its commercial-scale cellulosic biofuel plant in Mississippi. In Europe, EU policy requires that every Member State obtain 10% of its transport fuel from renewable sources by 2020, however, new legislation limits the contribution of biofuels derived from sugars, starch, and oil crops due to sustainability concerns, which are mainly about indirect land-use change. This, in combination with amendments to some national biofuel policies, has raised uncertainty among producers. So development of advanced biofuels has lagged due to the lack of EU-wide policy support, although some Member States have started to enact national policies, for example Italy announced a mandate of 0.6% advanced biofuels by 2018 [37].

Cellulosic biofuels may use crop residues or other wastes, but most plans for these biofuels rely on planting and harvesting fast-growing trees or grasses. At least some direct competition with food is still likely because such trees and grasses grow best and are most easily harvested on relatively fertile lands already dedicated to crops. Using cropland to grow trees and grasses rather than food crops for biofuels will probably not reduce, let alone eliminate, competition for cropland. Trees and grasses will have a hard time producing more biofuels per hectare than today’s crop-based biofuels. Alternatively, cellulosic biofuels might rely on harvesting existing forests or producing fast-growing trees or grasses on the world’s grasslands or woody savannas. Some researchers also point to abandoned farmland as a candidate for bioenergy production that avoids competition for land. But abandoned farmlands typically regenerate into forests, woodlands, or grasslands if left alone, which provide climate benefits that are already assumed and counted in climate change assessments. These benefits would be sacrificed by using that land for bioenergy. Although there is capacity to increase plant production on each hectare by increasing yields or by enhancing use of lands that are degraded, however, that capacity is already needed to meet rising demands for food and wood products while preserving ecosystems and their carbon. There are some biomass feedstocks that avoid the competition for land, namely various forms of wastes and residues. In the long run, such wastes might contribute modestly toward replacing some uses of fossil fuels. Phasing out the dedicated use of land to generate bioenergy, particularly biofuels, would reduce the food gap and, perhaps even more importantly, keep it from greatly expanding [38].

Searchinger and Heimlich [38] defined the dedicated use of land for bioenergy as the production of bioenergy that sacrifices alternative outputs from land. This narrow definition treats bioenergy production in isolation. For instance, some feedstocks (such as soybeans and rapeseed) for biofuel production would not fit into this definition because the land produces protein as animal meal as the main product and, secondarily, oil (either as cooking oil or a biofuel feedstock as biofuel products). That is, biofuel production from oil would not cause dedicated use of land for bioenergy production. The authors certainly did not promote bioenergy production from those feedstocks. Also, “land” can be very different in terms of suitability and productivity for different vegetation types. Land that may not be suitable for row crops could be suitable for growth of other vegetation types because of differences in nutrients, water, climate, and other requirements. Further, the authors treated marginal/degraded lands as not existing [39].

Searchinger and Heimlich [38] correctly pointed out that directing wastes like crop residues and municipal solid waste to biofuel production is a good use of these resources. They raised concerns, however, that dedicated cellulosic crops are not a promising option for a biofuel feedstock because they require land and do not have sufficiently high yield. Some of the studies on this topic indeed indicate that doubling of currently low cellulosic biomass yields is achievable [40]. Reliable reports indicate that significant areas of marginal lands exist that could be used to produce cellulosic crops that are currently underutilized [41]. Many studies have identified land where cellulosic biomass can be grown to avoid competition between cellulosic biomass and food production [42,43].

Conventional biofuels are currently produced in many countries and are based on well-known processes and feedstock. Apart from sugarcane ethanol, conventional biofuels will hardly be sustainable in the future as large-scale production would take away feedstock and land from food production and forestry. In addition, they are rather expensive and offer only limited reductions in greenhouse gas (GHG) emissions compared to fossil fuels.

Advanced biofuels promise to be more sustainable, with higher emissions reductions. They are based on biomass resources and land not used for other primary needs, such as food production and farming. Feedstock includes lignocellulosic residues from agriculture and forestry, fast- rotation non-food crops (possibly grown on marginal, non-arable land), organic fraction of urban waste and micro-algae. The conversion of these resources into biofuels requires processes that are currently under commercial demonstration or under development, with small plants in operation and large plants under construction or planned all over the world.

#### 2.2.1. Giant Reed for Biofuels

Non-food energy crops, such as woody plants (poplar, willow, *Eucalyptus* sp.) or herbaceous (*Miscanthus* sp., switchgrass or giant reed), is produced for bioenergy, biogas and biofuel purposes [44,45]. *Arundo donax* L. (common name “giant cane” or “giant reed”) is a perennial, rhizomatous species which has been introduced around the world by humans as an ornamental/crop plant. Giant reed is a sterile plant without any viable seeds, it can be propagated vegetatively from the rhizome or stems [10,35]. Propagules can be produced also by hydroponic or *in vitro* micropropagation methods [36]. Among the promising biomass plants, *A. donax* has high biomass yield per hectare and can be adapted to different types of soil and water condition [31,46]. It has lower agronomic input requirement than traditional crops [47]. It can be a good candidate to restore and recover soil ecosystem [48,49] and has ability to remediate the contaminated soil and water [50,51]. There is increasing commercial demand for giant reed production [9,10], however, there are limited data available about the production of biogas or bio-ethanol from giant reed.

From the second half of the 1990s, giant reed is regarded as one of promising plants of the biomass industry due to high biomass production per hectare [10,37]. For example in Central Italy, a 12-year field trial without irrigation could produce 38 tonnes dry matter per hectare per average year [38] and 20 tonnes dry matter with no fertilization on sandy soil [39]. For its cultivation low agronomic and energetic input is required [10,40]. Due to the high biomass per hectare and chemical composition of giant reed (Table 2.) large amounts bio-ethanol can be produced. Williams *et al.* [49] and Jaradat [50] reported same bio-ethanol production (11,000 L·ha^−1^) in case of 45 tones ha^−1^ biomass yield. According to Corno *et al.* [14] 12,960–15,228 L·ha^−1^ bio-ethanol can be produced, which is higher than reported from other energy crops for example than *Miscanthus x giganteus* [31].

Despite increasing interest in giant reed, propagation and production systems should be optimised reducing costs of bioenergy and bioproducts production to enhance economic feasibility. For biofuels production, it is necessary to improve sugar yield and reduce lignin content, therefore there have been more studies concerning various pretreatments of giant reed. Energy inputs and efficiency of converting giant reed to bioenergy and bioproducts are significantly influenced by the composition characteristics, such as minerals, cellulose, hemicelluloses, lignin, moisture content and recalcitrant compounds. It can be cultivated almost in all climatic zones. Cold seems to be a limiting factor, therefore researchers have started to develop cold-resistant giant reed varieties [36,41]. It has invasive potential in Mediterranean or warmer climates, so in case of establishment of giant reed plantations surrounding water conservation areas have to be avoided to reduce potential hazards.

#### 2.2.2. Algae for Biofuels

Algae have been cultivated commercially since the 1950s, mainly for the pharmaceutical industry, but have only recently gained attention as a potential source of biomass. Third-generation biofuel usually means biofuel from algae. To date, there have been numerous studies of algae and other water based biomass in order to identify strong candidates for biomass accumulation rates as well as lipid content for production of biodiesel. Marine and aquatic biomass can be a useful alternative source of biomass that can be used to produce a wide range of biofuels for commercial use. Primarily, growth of algae for the production of oils and energy conversion has focused on microalgae. Algae have potential as a feedstock for biofuels. Depending on their composition, different algae species may be suitable for a range of biofuels. Algae promise a potentially high productivity per hectare, could be grown on non-arable land, can utilise a wide variety of water sources (fresh, brackish, saline and wastewater), and potentially recycle CO_2_ and other nutrient waste streams. However, algae cultivation faces several challenges, related to availability of locations with sufficient sunshine and water, required nutrient inputs, and oil extraction [58].

Algae feedstocks for alternative fuels production are not economically competitive with fossil fuels at the present time. Furthermore, it has not yet been demonstrated that algae production systems offer improved sustainability characteristics. Like fossil fuel, algae fuel releases CO_2_ when burnt, but unlike fossil fuel, algae fuel and other biofuels only release CO_2_ recently removed from the atmosphere via photosynthesis as the algae or plant grew. Additionally, algal biomass productivity per hectare could eventually be higher than for terrestrial energy crops. Based on the high level of innovation demonstrated within the algal biofuels industry in just the past decade, it is likely that new technologies will continue to be introduced in the future. In fact, the introduction of these innovations will be critical if the sector is ultimately going to achieve commercial success [59,60].

Growth of aquatic and marine biomass is not without challenges because maximum growth rates of the microorganisms typically occur under very specific conditions. Furthermore, open pond algal systems are susceptible to contamination from various airborne microorganisms that can decrease overall productivity. But the main concern is the ability to separate algae from water, which due to their very dilute nature, can be expensive and inefficient. However, it is feasible to use algae as a means for tertiary wastewater treatment in order to utilize trace nutrients such as phosphorous- and nitrogen-containing compounds, or can be used at industrial processes as a way to absorb carbon dioxide by entraining algal cultures to gaseous exhaust streams [61].

Photosynthetic microorganisms such as microalgae and cyanobacteria could serve as an attractive feedstocks because they have higher growth rates requiring much less land area compared to plants and they can thrive in areas that cannot usually support mainstream agriculture. Algae can be cultivated at sea or on non-arable land, so there is no competition with current food production [62]. However, despite significant progress, reliable and cost-effective production of lipid- and protein-rich algal biomass have not been demonstrated at scales > 10 m^2^. Productivity and cost remain the two fundamental barriers to commercialization [63]. Commercially viable production of biofuel from algae will depend on effective strategies to generate high-volume, low-value biofuel along with high-value co-products.

Several companies and government agencies are funding efforts to reduce capital and operating costs and make algae fuel production commercially viable. Commercial cellulosic biofuel production began in the US in 2013, while algae biofuels are not yet produced commercially [6,37].

Recently, microalgae have emerged as a source than can play the dual role of bioremediation of wastewater and generation of biomass for biodiesel production. Growing microalgae on different types of wastewaters has been studied over the past decades. The success of such studies depends on the performance of the selected microalgae strains [64]. Moreover, the use of wastewater treatment becomes an economically attractive alternative [65]. Various types of algal biofilm reactors integrated with wastewater treatment were developed to overcome current limitation of algal biofuel production. So far the integration of algal bioreactor and wastewater has been limited to municipal wastewater while only a few agricultural wastewater have been used for algal bioreactors. However, algal biofilm reactors integrated with wastewater would have high potential for high productivity of algal biomass for biofuel and efficient wastewater treatment if various conditions are optimized [66]. In addition, a prospective life cycle assessments (LCA) of two algal systems were studied. The LCA impact results conclude that algal system producing biodiesel, animal feed, and succinic acid could be beneficial to the environment compared to that algae system that produces biodiesel and animal feed. Thus algae system could be a potential alternative or additional renewable system to mitigate environmental impact [67].

### 2.3. Feedstocks and Co-Products of Biofuel Production

Sugarcane is the predominant feedstock for ethanol production in tropical regions (Brazil). In temperate areas, ethanol is mostly made from cereals (maize in the USA and China, wheat in the EU and China). Main biodiesel feedstocks are soybean (Brazil, USA), rapeseed (EU), and oil palm (Indonesia and Malaysia). There are other feedstocks of minor importance, such as castor beans in Brazil, sunflower in the EU and jatropha in Mozambique, but these are not included in the analysis. Crop yield is high for sugarcane (Brazil, South Africa), sugarbeet, and oil palm. Cereal yields are high for corn in the USA, but less so for corn and wheat in the EU and China. Rapeseed and soybean yields are modest. Ethanol yields are highest for sugarbeet, and sugarcane (Brazil). Highest biodiesel yields were observed for oil palm (Indonesia, Malaysia). Of the four largest sources of biofuels: maize, sugarcane (bioethanol), soybean and rapeseed (biodiesel), only sugarcane appears to have a secure future although in the case of maize this will depend on the rate of yield improvement that could be achieved. The progressive divide between increase in demand and increase in production suggests that maize ethanol will lose long-term economic viability, unless the already high rate of yield per hectare improvement can be accelerated yet further. With the exception of oil palm, the yields of biodiesel crops (soybean and rape/canola) are too low to contribute significantly to future energy supply. Soybean and rapeseed produce far too little fuel per unit land area to remain competitive without mandates and subsidies. However, breakthroughs in engineering accumulation of oil in vegetative tissues may provide an alternative with the potential of developing a sugarcane that accumulates oil in place of sugar [68].

The bioethanol share in total grains demand—*i.e.*, corn, wheat and other coarse grains—is about 6% of global production. The fuel ethanol sector accounts for 13% of global maize consumption and 20% of global sugar cane production. An estimated 143 million tonnes of grain is used globally for ethanol. The US is the global leader in grain ethanol production, accounting for roughly 90% of total grain use for ethanol, followed by the European Union, China and Canada. Maize accounted for the majority of grain use for ethanol in the United States and China. European and Canadian producers principally use wheat and maize for ethanol. About one-third of the volume of grain processed for ethanol was used to produce animal feed as co-products, thus, the equivalent of two-thirds of the of grain were used to produce fuel. Feed market impacts of increased maize use for ethanol are smaller than that indicated by the total amount of maize used for ethanol production because of DDGS. While ethanol expansion raised demand for maize, DDGS partially offsets the impact on the feed market. Consequently, the net effect in the domestic feed market of a tonne of maize being used for ethanol production is less than a tonne. For example, the amount of feed (corn and soybean meal) replaced by the DDGS represents about 38% (weight basis) of the maize used in the associated ethanol production process for a given crop year. Furthermore, grain use for ethanol is expected to moderate in accordance with slowing national mandates in the US and the EU [4].

The biodiesel share of rapeseed, soybean and palm oil demand is around 11% of global vegetable oil production. The share of waste biodiesel feedstocks such as animal fat and used cooking oil increased to 15% in total biodiesel output. Continuously growing demand for protein meal has been the main driver behind the expansion of oilseed production in recent years. This has increased the share of protein meal in the value of oilseeds and favoured soybeans over other oilseeds. Compared with coarse grains and other feed ingredients, protein meal prices have stayed relatively high. Global oilseeds production reached 530 million tonnes (soybean 320 and rapeseed 70 million tonnes) in the 2014/15 marketing year. At the same time soybean production increased faster than production of rapeseed, sunflower, increasing the sector’s concentration. Vegetable oil production increased to 180 million tonnes (out of this 60 million tonnes palm oil). Demand growth has slowed in recent times due to stagnating biodiesel production from vegetable oils in developed countries. Production of rapeseed in Canada and the European Union is expected to grow much slower than in the previous decade as high oil-containing oilseeds like rapeseed are more affected by the slower growth in vegetable oil prices [7]. Vegetable oil use in biofuel production account for about 20 million tonnes a year. Soybean and rapeseed oil has a 70% share of the total feedstocks used in biodiesel production worldwide. Oilseeds such as rapeseed or canola and soybeans represent the most common source of vegetable oil feedstocks for biodiesel production. An estimated 6 million tonnes of rapeseed oil and 7 million tonnes of soybean oil is used globally in the production of biodiesel, representing roughly 70% of the total feedstocks used in global biodiesel production [6,7].

Biofuel production from wheat, maize, rapeseed and soybean yields valuable protein-rich co-products such as rape meal, soymeal and dried distillers' grains and solubles, which can be used as animal feed in the livestock industry. Because protein-rich crops generally require a relatively large amount of land for a given output compared with cereal crops, the use of co-products can reduce net land use. Co-products considered in this study include dried distillers’ grains with solubles (DDGS), soy meal, rapeseed meal. Palm kernel cake and sugar beet pulp is an important, though low quality component of feed concentrates. These co-products are produced in insignificant amounts and are therefore not considered in this analysis.

#### Animal Feed Produced from the Ethanol and Biodiesel Industry

Over 90% of the ethanol produced today comes from the dry milling process and 10% from wet milling. Both the wet and dry mill processes utilize only the starch portion of the corn kernel for ethanol production. The remaining protein, fat, fiber and other nutritional components remain available for use as animal feed. In distillers dried grains with solubles (DDGS) or distillers dried grains (DDG), these remaining nutritional components from the corn kernel are essentially concentrated by a factor of three, meaning typical distillers grains have at least three times as much protein and fat as an equivalent amount of corn. If the distillers grains are being fed to livestock in close proximity to the ethanol plant, the drying step can be avoided and the product is called wet distillers grains (WDG). An estimated 85% of existing dry mills have the capability to extract corn oil, which is then sold as an individual feed ingredient or as a feedstock for biodiesel production. In the wet milling process corn oil from the germ is either extracted on-site or sold to crushers who extract the corn oil. The remaining fiber, gluten and starch components are further segregated and sold as corn gluten feed (CGF) or corn gluten meal (CGM). The remaining starch can then be processed in one of three ways: fermented into ethanol, dried or modified corn starch, or processed into corn syrup.

In fact, one-third of every bushel of grain that enters the ethanol process is enhanced and returned to the animal feed market, most often in the form of distillers grains, corn gluten feed and corn gluten meal. These nutrient-dense co-products are fed to beef cattle, dairy cows, swine, poultry, and fish in nations around the world. In the 2013/14 marketing year, the U.S. ethanol industry produced an estimated 39 million metric tonnes, the EU 4 and China 2 million metric tonnes of high-quality feed, namely 41 million tonnes of distillers grains and around 4 million tonnes of gluten feed and gluten meal. Estimated average potential U.S. DDGS feed consumption is around 62 million tonnes a year, an amount much higher than the current DDGS supply [16,18]. Corn gluten feed and meal production has remained relatively constant in the last decade, as the majority of growth in ethanol production has come from dry mills. As for the wet milling co-products, corn gluten feed is primarily fed to dairy and beef cattle, while corn gluten meal is used to feed a wide variety of species, including poultry and fish. Corn gluten meal, which features a very high concentration of protein, is often used as an ingredient in pet food as well. While much has been learned over the past decade about the benefits of feeding co-products, much more research is under way at public and private institutions to increase co-product inclusion levels to best take advantage of their nutritional and economic benefits. While co-products of the ethanol industry are used by the domestic markets in the EU and China the United States exported around 10 million tonnes of distillers grains and approximately 2 million tonnes of corn gluten feed and corn gluten meal—roughly 28% of total U.S. production—in to nearly 50 countries worldwide in 2013. These exports in 2013 were volumetrically equivalent to about 24% of total 2013/14 corn grain exports. Increases in ethanol co-product exports are supplementing corn export levels, meaning total exports of corn and corn products continue to trend upward [69,70].

By economically displacing traditional feed ingredients, ethanol co-products effectively reduce the livestock and poultry industry’s demand for maize and protein meal. The return of co-products to the feed market has agricultural land use implications as well; at least one-third of every hectare “dedicated” to ethanol production should actually be characterized as producing feed, not fuel. Co-products from grain ethanol production are an increasingly important and valuable component of the biofuels sector and the global feed market. Today, more than 85% of existing dry mills have the capability to extract corn distillers oil (CDO) in the U.S., which is then sold as an individual feed ingredient or as a feedstock for biodiesel production. Distillers grains are increasingly recognized as an extremely efficient feed source. In many cases, co-products are a more effective source of energy and protein than the ingredients they are replacing in the diet. Distillers grains provide approximately 130%–150% of the energy of an equivalent amount of corn when fed to beef.

Historically, distillers grains have generally sold at a significant discount to competing feed ingredients, meaning they are a good value for livestock and poultry feeders around the world. Over the past several years, DDG prices have consistently sold for 75%–90% of the Central Illinois corn price and 40% of Central Illinois soybean meal prices. However, in 2011 and 2012, DDGS prices sold for 83% the price of corn in the Central Illinois reference market but just 47% the price of soybean meal. Moreover, as prices for soybean meal and other protein feeds spiked in 2013 due to the 2012 drought, DDGS was increasingly used as a replacement for protein meals. Thus, DDGS prices have averaged 109% the price of corn and 40% the price of soybean meal since the beginning of 2013 until mid-2014. A tonne of DDGS contains approximately 270 kilograms of protein, while a tonne of high-protein soybean meal contains 436 kilograms of protein. Thus, DDGS served as a lower-cost means of meeting protein needs during the period in which the 2012 drought affected global feed supplies [69].

Feed co-product output is expected to grow more slowly in the coming years. However, a number of new and emerging technologies may change the composition and further improve the quality of feed co-products. As discussed earlier, about 85% of dry mills today extract CDO from the stillage on the back end of the process. This CDO can be sold into the feed market (particularly for poultry) or used as a feedstock for biodiesel. When used as a biodiesel feedstock, CDO displaces higher value fats and vegetable oils that are typically used in food or feed applications. The feed co-products resulting from oil extraction practices typically have lower fat and higher protein content than conventional distillers grains. These “reduced fat” co-products can be more useful for certain species. Dry fractionation is another technology that may emerge more broadly in the dry mill ethanol industry. Essentially, this practice allows dry mill ethanol producers to separate the corn germ and other components from the starch on the front end of the ethanol process. The germ can then be processed and sold as feed or as feedstock for further processing for other uses. Further, some dry mill facilities are examining the potential of converting corn kernel fiber to cellulosic ethanol. This process would reduce the fiber content of DDGS, which may make the product more palatable at higher inclusion levels for monogastric animals. Additionally, a number of new and emerging ethanol processing aids are likely to improve the nutritional quality and utility of ethanol co-products. The success of these new technologies will depend on the demands of the ethanol industry’s customers in the animal feed market. New technologies and practices promise to change the complexion of the ethanol co-products market in the years ahead.

The main components used to make biodiesel are soybean or rapeseed oil and an alcohol source, typically methanol. Seed meal co-products are left after oil is extracted from soybeans and canola oil. An estimated 80% of soybean seed 60% of rapeseed is left from the extraction process as seed meal, creating a significant quantity of this important co-product. The oilseed meal can be used immediately as an animal feed without further treatment. About 7 million tonnes of soybean oil and 6 million tonnes of rapeseed oil is used in biodiesel production contributing to almost 3.6 million tonnes of rapeseed meal and 5.6 million tonnes of soybean meal output [6]. Taking into consideration that 210 million tonnes of soymeal and 40 million tonnes of rapeseed meal is produced a year globally, the co-products of biodiesel production have a moderate impact on the feed market. The main direct by-product of biodiesel production is glycerine constituting about 10% of these materials on average. There is a limited demand for glycerin, the by-product of biodiesel production for a number of food, beverage, personal care and oral products, as well as pharmaceutical and other industrial uses. Glycerin can be used effectively in livestock rations to replace fossil-based glycerine. Importantly, glycerine can also be utilized as a feed ingredient for livestock rations. Crude glycerin contains similar energy to that of maize for pigs [24].

### 2.4. Attributes of Co-Products of Biofuels Production

The biofuel production process produces the fuel and other co-products. The type and quantity of co-products strongly depends on the biofuel production chain. The economic viability of the biofuel industry depends to a large extent on the ability of the industry to derive value from the biofuel it produces as well as the co/by-products that are generated during the process. When co-products are used credits can be attributed to the biofuel production chain. Credits include GHG emission savings, avoided land use or avoided energy use. The challenge is to decide how to distribute quantities of potential credits between the fuel and the co-products.

Credits for co-products are an important element in the calculation of greenhouse gas (GHG) reductions of the different biofuel production chains compared to fossil fuel use. For the evaluation of GHG emission savings common methods include mass based (allocation by mass), market value-based (allocation by price), and energy content-based (allocation by energy content) calculations. For reasons of feasibility the EC recommends in its regulatory framework of the proposal for a directive on renewable energy targets to apply allocation by energy content to determine GHG savings compared to fossil fuels [71]. However, for a fuel supplier it is not possible to know for certain what the substituted product really is and thus GHG emissions of these substituted products are uncertain. Moreover co-products used as animal feed can replace many different animal feed products, which have differing GHG performances.

Co-products can be applied as animal feed, substituting other feeds, such as maize and soymeal. When for example maize and soymeal is substituted, less land for feed cultivation is needed. This substitution reduces the indirect land use and therefore the impact of indirect land use change and intensification is substantially. The impact of the co-products on land use can be included. Recently several studies have attempted to include attribution of those co-products, which can be used as animal feed in an analysis of land use requirements of biofuels. When co-products are used for heat and process energy the energy balance improves. In those cases, feed has to be produced elsewhere and even ILUC effects cannot be excluded [1].

#### 2.4.1. Economic Implications

Biofuels support policies in OECD countries are costly and there are alternatives to current support policies for biofuels that would more effectively allow governments to achieve their objectives. The impact of biofuels policies on GHG emissions is limited, however, biofuels support policies have significant impacts on global commodity prices. Alternative policy approaches may offer greater benefits including reduced energy demand and GHG emissions, and freer trade in biofuels. Further research can contribute to second generation biofuels from more efficient feedstocks, such as cellulose and other biomass [72]. Replacing fossil fuels with biofuels has the potential to generate a number of benefits. Biofuels can be produced domestically, which could lead to lower fossil fuel imports. Reducing the demand for petroleum could also reduce its price, generating economic benefits for domestic consumers, but also potentially increasing petroleum consumption abroad [73]. Bioenergy could enable farmers in poor regions to introduce agricultural technologies and improve infrastructure to increase farm productivity and thus to raise farmers’ income [74]. Bioenergy and food production can co-exist and enhance each other by advancing technologies and increasing yields [75], thus complementing each other instead of competing against each other.

Feedstock generally accounts for around 70% of production costs, with processing, transportation, and other costs making up the remainder. Therefore, declining feedstock prices helped industry by reducing overall production costs. In 2014, most fuel ethanol was produced from sugar crops (roughly 61%), with the remainder from grains (roughly 39%). Feedstocks vary significantly depending on the country or region. For example, fuel ethanol production in the United States is based largely on corn, whereas Brazil relies primarily on sugar crops, and China on sweet sorghum, cassava, and other non-grain crops. Global biodiesel production is based largely on vegetable oils, mostly from rapeseed (Europe) and soybeans (United States, Brazil, Argentina), with smaller shares from palm (Indonesia) and other sources such as jatropha and coconut. Biodiesel production also includes industrial by-products such as used cooking oils (the main feedstock in China) and animal fat. In Europe, the relative share of cooking oil and tallow in biodiesel production is increasing as EU policy allows these feedstocks to be double-counted in transportation targets [37].

Increased acres for corn farming in the U.S, are driven by the significant increase in corn prices since 2005. However, several key factors cause increases in corn price, including U.S. corn ethanol production, weather events, recent grain demand increases, and diet changes in emerging economies [76]. Economic models show that biofuel use can result in higher crop prices, though the range of estimates in the literature is wide. Projections for the effect of biofuels on corn prices in 2015 ranging from a 5 to a 53 percent increase [77]. The possible impact of developed countries’ biofuels policies on global food prices became a significant concern in 2007, when global grain prices reached historic heights. Though some experts associated the unprecedented price spikes in food grain and oilseed with these countries’ biofuels policies [78,79,80], most of them now agree that these policies are unlikely to have been the main culprit, although they may have been a factor emphasizing that biofuel policy is only responsible for part of that fraction of price increases in food grain commodities that is due to biofuels [81].

Another study estimates that the impact of EU biofuels demand from 2000 until 2010 has increased world grain prices by about 1%–2% and oilseed prices by around 4%. It also estimates that without any cap on crop-based biofuels, EU policy could raise grain prices by 1%, and oilseed prices by 10% by 2020 [82]. Increasing the productivity of current and emerging bioenergy crops per unit land area is not only critical to economic viability, but also to biodiversity by minimizing the total land area needed. Land sparing is found far more effective than land sharing in strategies to realize bioenergy. Maize ethanol, often portrayed as the villain of the piece in the food *versus* fuel debate, may in fact have been key in stimulating yield improvement, including through genetically modified (GM) traits, that has resulted in increased exports of grain from the USA while providing a buffer in drought years [69].

Concerns over the role of food crops in the energy sector are well-founded, and for this reason (among others), the Renewable Fuel Standard caps corn ethanol volumes at 15 billion gallons. In short, for biofuels to expand their role in the transportation sector in the United States, they must be produced from cellulosic feedstocks, such as energy grasses, short rotation woody crops, crop residues, wastes, and other sources. In the EU new legislation limits the contribution of biofuels derived from sugars, starch, and oil crops due to sustainability concerns, which are mainly about indirect land-use change.

Biofuel co-products often substitute for higher priced feeds in animal rations. The increased use of agricultural commodities for biofuels has led to higher costs for animal feeds, however, increased substitution of co-products for traditional feedstuffs in feed rations mitigate input cost increases faced by livestock and poultry producers. Growth in the use of agricultural commodities for biofuels is expected to continue in the next 10 years, but with growth rates slowing in key producing countries as government-imposed limits on grain use for biofuels are reached and new non-agricultural feedstocks are commercialized [4,5].

DDGS and corn prices are highly correlated, and their correlation has strengthened in recent years. Soy and rapeseed meals have always been a major component of animal feeds, because they are excellent sources of protein. The impact of increased ethanol production is largely felt through competing crops as increased production of DDGS and oilseed meals can jointly reduce the demand of livestock industry for maize and oil meals and offset the increase in the demand of ethanol industry for grains due to the US and EU biofuel mandates. With increasing biofuel production the production of these co-products also increases. Prices of co-products are highly correlated with prices of feedstocks, such as grains and oilseeds and they represent an important component of total industry revenues. As a result, co-products prices fall relative to other feed ingredients. This encourages livestock producers to use more biofuel co-products in their production processes. On the other hand, any reduction in the prices of co-products diminishes total revenue and acts as a brake on growth of the biofuel industry. Biofuel co-products function as both a shock absorber and a price adjuster [83].

Between 1983 and 2006 the price of DDGS relative to maize has fallen by nearly 50%. This has provided a strong incentive for livestock producers to use more DDGS in their production process and has also enhanced US exports of DDGS [83]. The ratio of the average price of DDGS to the average price of maize reported for Iowa plants from 2007 through March 2015 ranged from 0.67 to 1.48 and averaged 0.91 for the entire period [84]. Further, the relative contribution of distillers’ grains to gross returns has varied over time as the price of DDGS has varied.

The net contribution of DDGS to net returns for ethanol production is a function of the ratio of the price of DDGS to the price of maize. When the ratio is high, distillers grains provide a larger contribution to net returns and vice versa. With all other prices unchanged, a decline in the price of DDGS would result in a corresponding decline in net returns. For example, the current high ratio is contributing to net returns of ethanol production [84].

The global grain and oilseed supply has grown substantially in recent years and increased use of these commodities for biofuels production has not led to reduced availability for feed or food use. The amount of grain and oilseeds available for uses other than ethanol and biodiesel production is expected to grow more significantly in the long term, as feedstock use for biofuel production moderates in accordance with slowing national mandates.

#### 2.4.2. Environmental Implications

##### Land Use Change and GHG Emission

Changes in land use, principally those associated with deforestation and expansion of agricultural production for food, contribute about 15% of global emissions of GHG. Currently, less than 3% of global agricultural land is used for cultivating biofuel crops and land use change associated with bioenergy represents only around 1% of the total emissions caused by land-use change globally most of which are produced by changes in land use for food and fodder production, or other reasons [85]. Indirect land-use changes, however, are more difficult to identify and model explicitly in GHG balances. Most current biofuel production systems have significant reductions in GHG emissions relative to the fossil fuels displaced, if no indirect LUC effects are considered.

Despite the availability of additional land, however, land use in agriculture has actually grown very slowly for decades. The best rainfed cropland is already being used, and expansion to other areas would incur higher input costs on average. Existing land is being used more intensively in most regions, as the practice of multiple cropping has spread, particularly in areas where land is relatively scarce, such as in Asia. Rising concerns and government regulations over climate change, biodiversity and resource management will push agriculture towards greater sustainability and lower environmental costs while competition between food and biofuels could also become more intense [86].

Changes in land use patterns may increase GHG emissions by releasing terrestrial carbon stocks to the atmosphere [87]. Biofuel feedstocks grown on land cleared from tropical forests, such as soybeans in the Amazon and oil palm in Southeast Asia, generate particularly high GHG emissions [88]. Even use of cellulosic feedstocks can spur higher crop prices that encourage the expansion of agriculture into undeveloped land, leading to GHG emissions and biodiversity losses [89].

Although biofuel production is only one of numerous contributors to land-use change, Fritsche and Wiegmann [90] estimate that by 2020 the overwhelming majority of land-use change—both direct and indirect—could be caused by feedstock production for biofuels. Because of its indirect nature, ILUC is a not a local phenomenon that can be observed in a given place and time. It cannot be monitored for individual feedstocks (e.g., biofuel crops), because production can be displaced anywhere in the world and because displacement can be distributed through global trading or occur with significant time lags.

Depending on the location of production, the total amount of area converted as well as the GHG emissions per hectare converted can vary greatly. And this global distribution of production depends on the assumptions made about the role of geography in international trade. Assumptions about yield response on the extensive and intensive margins are certainly critical in determining land-use change outcome and trade elasticity. Yield response for other commodities (other than the focus feedstock) and other regions are even more important than the yield response of the specific feedstock/region under examination (U.S. corn in this case). In addition to the intensive margin of yield response, changes in the assumption about crop yield response at the extensive margin can also have a dramatic impact on the resulting net global cropland requirement. Furthermore, consumer demand elasticity for food products play a critical role in determining the necessary expansion in global land-use following expansion of ethanol production [11].

The demand for land to produce bioenergy feedstock can cause shifts in land use patterns that can affect agricultural land. Land use change (LUC) impacts of biofuels have been a concern since Searchinger and Heimlich [87] first published a paper investigating LUC associated with bioenergy crop production and subsequent GHG emissions. Since that time, understanding of biofuel-associated LUC has improved greatly because better land use data are available and economic models for modeling LUC have been improved significantly. Recent LUC GHG results indicate that Searchinger and Heimlich [87] originally over-estimated LUC GHG emissions significantly [91].

Later, Searchinger and Heimlich [38] maintained that additional biomass for bioenergy is the key for bioenergy-derived GHG reductions. They further asserted that double counting of carbon occurs in analyses of bioenergy because the biomass additionality issue is not addressed. The concept of additionality for GHG reductions really asks what the counterfactual scenario is when bioenergy does not exist or exists only at current levels. The authors concluded that any dedicated biomass growth would result in double counting of biomass by assuming that biomass for bioenergy is grown on the lands where biomass would be grown anyway and with the same yields with no differences in land productivity of biomass between the two scenarios. Land biomass productivity differences could be caused by biomass yields, changes from single cropping to double cropping (or even triple cropping) because of bioenergy production [92], and the ability to use marginal/degraded lands for bioenergy production. Careful characterization of counterfactual scenarios and bioenergy scenarios has been conducted to address the biomass additionality issue. In fact, some analyses point to the need to compare biofuels to marginally produced, high-GHG-intensity fossil fuels [93].

According to Searchinger and Heimlich [38] phasing out bioenergy that uses crops or that otherwise makes dedicated use of land is a sound step toward a sustainable food future. However, U.S. farm acres for different crops illustrate that while corn acreage has increased in parallel with the build-up of the corn ethanol industry between 2004 and 2013, total principal crop acreage has remained fairly constant in the U.S. [94]. These observed trends are consistent with Taheripour and Tyner [95], who did observe crop shifting (e.g., wheat fields converted to corn agriculture) in the United States in this time period as a key mechanism for additional corn production. Another mechanism is likely the conversion of grasslands, wetlands, and other lands. The key point is that agricultural acreage in the United States has not significantly increased despite a dramatic biofuel boom. Additionally, the U.S. Renewable Fuel Standard states that biofuel feedstocks must come from land that was not forested before 2007. This provision limits the expansion of agricultural land into forested lands for biofuel production. In the case of woody feedstocks, the forests from which they derive must have been managed plantations before 2007.

Reduced deforestation and increased agricultural production can occur simultaneously in tropical forest frontiers, provided that land is available and policies promote the efficient use of already-cleared lands (intensification) while restricting deforestation. It remains uncertain whether government- and industry-led policies can contain deforestation if future market conditions favor another boom in agricultural expansion [96].

Academic studies using economic models have also found that biofuels can lead to reductions in lifecycle GHG emissions relative to conventional fuels [73,97]. Second and third generation biofuels have significant potential to reduce GHG emissions relative to conventional fuels because feedstocks can be produced using marginal land. Moreover, in the case of waste biomass, no additional agricultural production is required, and indirect market-mediated GHG emissions can be minimal if the wastes have no other productive uses.

Biofuel production and consumption, in and of itself, will not reduce GHG or conventional pollutant emissions, lessen petroleum imports, or alleviate pressure on exhaustible resources. Biofuel production and use must coincide with reductions in the production and use of fossil fuels for these benefits to accrue. These benefits would be mitigated if biofuel emissions and resource demands augment, rather than displace, those of fossil fuels.

Biofuel production and processing practices can also release GHGs. Fertilizer application releases nitrous oxide, a potent greenhouse gas. Most biorefineries operate using fossil fuels. Some research suggests that GHG emissions resulting from biofuel production and use, including those from indirect land use change, may be higher than those generated by fossil fuels, depending on the time horizon of the analysis [89,98].

Regarding non-GHG environmental impacts, research suggests that production of biofuel feedstocks, particularly food crops like corn and soy, could increase water pollution from nutrients, pesticides, and sediment [99]. Increases in irrigation and ethanol refining could deplete aquifers [99]. Air quality could also decline in some regions if the impact of biofuels on tailpipe emissions plus the additional emissions generated at biorefineries increases net conventional air pollution [99].

Biofuel co-products help mitigate the environmental consequences of expansion by the biofuel industry. For example, DDGS substitutes for both maize and soybean meal in livestock rations. This reduces the land use consequences of biofuel production and eases the demand for chemical inputs, such as fertilizers and pesticides, in crop production. By reporting only the gross usage of maize for ethanol, the implication was that all the maize going into ethanol production resulted in fuel ethanol [1]. According to the conventional assumption ethanol producers return a full one-third of the maize processed back to the feeding sector which is the difference between the gross and net volume of maize used for ethanol. However, in aggregate, a metric tonne of DDGS can replace, on average, 1.22 metric tonne of feed consisting of corn and soybean meal in the United States. In fact, the amount of feed (maize and soybean meal) replaced by the DDGS represents 38% a weight basis of the maize used in the associated ethanol production process for a given crop year. If co-products are taken into account the net use of feedstocks decline [18]. More complicated, but no less important, is the impact of DDGS on land use change and the GHG emissions associated with maize ethanol production. Most existing biofuel regulations significantly undervalue the contribution of DDGS when assessing the net GHG impacts of maize ethanol assuming that one metric tonne of DDGS replaces only one metric tonne of corn, with no substitution of soybean meal. The importance of DDGS is being undervalued by the regulatory agencies requiring a GHG assessment of ethanol. In the future accurate DDGS accounting is of increasing importance [1].

The proportion of global cropland used for biofuels is currently some 2% (30–35 million gross hectares) with wide differences among countries and regions. The review by [100] studies biofuel expansion between 2000 and 2010 in Brazil, the USA, Indonesia, Malaysia, China, Mozambique, South Africa plus 27 EU member states. In 2010, these countries produced 86 billion litres of ethanol and 15 billion litres of biodiesel representing 95% of global biofuel production. Land devoted to biofuel production was calculated at 32 million ha in 2010, an increase of 25 million ha as compared to 2000. According to [101] land required to produce biofuels grows from 30 million hectares in 2010 to 100 million hectares in 2050. Ignoring co-product generation in early biofuel impact assessments has led to an overestimation of land requirements, in most cases by 40% or more. The contribution of feed co-products is relatively high in the USA, China, and the EU due to the large share of cereals with high feed yields. It is low in Brazil where ethanol production is dominated by sugarcane which generates no feed co-products. Implications for land use will, however, also depend on the role of yield improvement [100]. Another report published in 2008 by CE Delft estimates that the use of co-products generated from rapeseed, soy, wheat and maize can reduce net land use by 11% to 25%. Biofuels produced from some feedstocks such as sugarcane, where nearly the entire product is used for producing biofuel, do not generate such co-products [102]. Co-products are supposed to be credited with the area of cropland required to produce the amount of feed they substitute. By adding co-products substituted for grains and oilseeds the land required for cultivation of feedstocks declines to 1.5% net land requirement of the global crop area. This substitution reduces the indirect land use and therefore the impact of indirect land use change and intensification is substantially [1].

In 2010 about 30–35 million gross hectares of feedstock was needed for fuel ethanol and biodiesel production. The proportion of global cropland used for biofuels is currently some 2% with wide differences among countries and regions. In the cases of grains and oilseeds, DDGS, CGF/CGM and oil cakes (mainly rapeseed and soybean cake/meal) substitute grain and soybean as feed. It means that not all the feedstocks used for bioethanol production should be subtracted from the supplies.

Based on the land-use efficiencies land use for biofuel production would need to increase from about 30–35 million hectares to around 100 million hectares in 2050. This corresponds to an increase from 2.5% of total arable land today to around 6% in 2050. This expansion would include some cropland, as well as pastures and currently unused land, the latter in particular for production of lignocellulosic biomass [2].

##### Algae as Animal Feed

Many studies have shown the suitability of algae as a potential animal feed and as a replacement for conventional protein sources such as soybean and fish meal. Unfortunately, the trend is to avoid using live algae due to their high cost and production difficulties. The use of micro-algae as animal feed is more recent. Algae biomass is a valuable feed supplement or substitute for conventional protein sources. The incorporation of algae into poultry rations offers the most promising prospect for their commercial use in animal feeding. Another growing market is the utilization of micro-algae in aquaculture [103]. In poultry, algae can be used as a partial replacement for conventional proteins with the incorporation of 51% [104]. Also, according to [105], they may serve as almost the sole protein source in laying hens, and in several countries, they are officially approved as chicken feed. In pigs ration, [106] assumed the incorporation of even 33%, without negative symptoms. According to another study the feed intake of the pigs on the algal meal diets was not affected until the third period of the experiment and suggests that the acceptance from a palatability point of view when added at 10% is not a major issue although the manipulation of the gut microbiome to algae meal is possibly of some concern although a larger number of piglets raised in commercial environments would be required to better define this as an issue [107]. However, it should be expected that the most suitable for feeding with algae are ruminants, because they are able to digest even an unprocessed algal cell wall [105]. Manufacturers of these organisms worldwide have recognized different potentials and therefore focused just on the food and feed industry [108]. Although, microalgae are eaten as a food in China and Chad and had been considered as a solution to the world’s food shortage, their use on a global scale appears limited to health food and food supplements [109].

##### New Market Opportunities for Biofuel Co-Products

The biofuels industry has evolved rapidly over the last three decades with developments in processing techniques and an expansion of the range of plants and other natural energy sources being considered as feedstocks. On-farm application of the co-products, on which the viability of the industry depends, is often ahead of unbiased research to support its use. Much of the potential research identified as needed is concerned with co-product feeding value, the need for standardization of products from within an individual plant and between plants, and the search for new feedstocks together with safety standards. However, the economic importance of biofuel industrial production chains will increase in the coming years and will be a promising source of co-products that are useful for sustainable farming systems, biomaterials applications and crop disease management.

A potential market for DDGS and oilseed meals that biofuel producers may consider is the fertilizer/agriculture market. DDGS and oilseed meal co-products can be effective fertilizer sources for plants. In addition to containing most macronutrients and micronutrients needed to support plant growth, these co-products have low C:N ratios, which means that they are rapidly decomposable and can release nutrients to plants in a timely manner. This property is of particular interest to organic agriculture markets, where nutrient sources and fertilizers containing enough readily mineralizeable N to impact yield are scarce. Competition with animal feed markets has prevented widespread adoption of biofuel co-products as fertilizers. DDGS and many oilseed meals hold more value as an animal feed than as a fertilizer source, therefore animal producers are willing to pay for feed materials based on feed value instead of the lower fertilizer value. However, fertilizer markets may become more appealing to biofuel producers due to over-production of DDGS, high costs of oilseed meals, DDGS feed quality issues, and interest from organic growers in alternative nutrient-rich fertilizer sources [110,111,112].

The growth of biofuel industries caused the generation of a huge amount of under-valued co-products of different types including DDGS, bagasse, lignin, protein-rich meals and crude glycerol. In order to claim biofuel production as the sustainable technology of future, it is necessary to create value-addition to these co-products. This also provides solution to the emerging issues on the environmental impact of the accumulation of these co-products. Furthermore, the technological development related to the value-added uses of biofuel co-products in biomaterials applications is expected to create new ecofriendly products and strengthen the bioeconomy. Among these co-products, DDGS, CGM and soybean meal are traditionally used for animal feed applications. However, recent researches show the huge potential of these co-products for biomaterial applications (polymeric biocomposites, thermoplastic, biobased films for food packaging applications *ect.*). Crude glycerol co-produced in biodiesel industry has huge opportunities in biomaterial applications including chemicals/monomers, plasticizer, hydrogen generation, carbon source for bacterial growth and polyesters production. Lignin, the major co-product of lignocellulosic ethanol can be used as polymer blends/composites, adhesives, and carbon fibres. In addition to that, these biofuel co-products also create opportunity for the fabrication of various nanostructured materials for the high value applications [113].

Moreover, the use of co-products from the biofuel production chains, especially for crop disease management, is an under-explored area in the research community. In recent years, a relevant number of studies for the control of plant diseases have been carried out on bio-chars, oil-less seed meals, suppressive composts that are derived from agricultural waste and exhausted biomass, and steam-exploded liquid waste. The co-products of particular interest in crop protection are oil-less seed meals and glycerin derived from the biodiesel chain, steam-exploded liquid waste derived from a 2nd generation bioethanol chain, and charcoal obtained from the pyrolysis of plant biomass. Biofuel chain co-products have great potential but sometimes give inconsistent disease control, which limits their use in crop protection. Nevertheless, the benefits of biofuel chain co-products outweigh their drawbacks, but the impact of this approach on pathogen populations and disease suppression is often unpredictable. Future research should mainly focus on the development of biofuel chain co-products that enhance the activity of beneficial microbes and improvement of the effectiveness of biofuel chain co-products via applications of multicomponent bio-fungicides [114].

## 3. Materials and Methods

The literature on the impacts of biofuel expansions is already substantial, however, the feed value of increasing biofuels co-products, which are supposed to be credited with the area of cropland required to produce the amount of feed they substitute have received much less attention.

The search platform Web of Knowledge and search engine Google Scholar are primarily used to collect the relevant literature. In addition, backward searches through bibliographies of academic studies and reviews as well as hand searching websites of academic projects and conferences on biofuel are also applied. Only literature in English is included in this paper so as to ensure accessibility. Since the rapid progress of this research filed, literature is also limited to the papers mainly published in or after 2010. The literature reviewed is selective and critical. Highly rated journals in scientific indexes are the preferred choice. We carefully select 114 papers which are considered as important or innovative studies, or comprehensive reviews offering us a big picture of biofuels and their co-products as livestock feed. The literature review is categorized into three topics:

1. Co-products of biofuels as livestock feed: biofuel’s co-products refer to co-products that are generated during the process and used as animal feed
2. Economic, environmental and land use implications of biofuel’s co-products. When co-products are used credits can be attributed to the biofuel production chain including economic viability, GHG emission savings and avoided land use. Impact on land use and GHG emissions are the main focuses the existing studies paid attention to.
3. Advanced biofuels: future production is influenced by the competition between conventional and advanced biofuels based on food crops and lignocellulosic feedstocks.

Amount of literature examined in this paper classified by topic:

**Table:** 

Topic	Amount
Co-products	14
Land use	26
Economics	18
Environment	13
Bioenergy potential	12
Conventional biofuels	11
Advanced biofuels	20

## 4. Conclusions

Estimates on impacts of biofuel production often use models with limited ability to incorporate economic and environmental implications by ignoring generation of co-products from biofuel production. Co-product generation in early biofuel impact assessments was ignored leading to an overestimation of land requirements and GHG emissions. The output of feed co-products is relatively high in the USA, the EU and China due to the large share of grains used in ethanol production with high feed yields. It is low in Brazil where ethanol production is dominated by sugarcane which generates no feed co-products. Co-product yields are low for rapeseed and soybean used in the biodiesel industry. By economically displacing traditional feed ingredients co-products from biofuel production are an important and valuable component of the biofuels sector and the global feed market. Moreover, the return of co-products to the feed market has economic, land use and GHG emissions implications as well. Models used to evaluate biofuel policies should be enriched by incorporating more and better information on changes in land use, and economic and environmental implications of co-products. This information should be considered in discussions on food, feed *versus* fuel debate and land-use change caused by biofuel policies.

Recent years have seen a tremendous increase in the production of biofuels from agricultural commodities. Food-crop based feedstocks are expected to continue to dominate ethanol and biodiesel production over the coming decade. Growth in biofuel production has been accompanied by increased output of animal feed co-products from common biofuel processes. Globally, these feed co-products are growing in volume and importance. While the increased use of agricultural commodities for biofuels is generally expected to contribute to slightly higher input costs for certain livestock and poultry feeds, the impacts are expected to be modest and can be mitigated in part by increased substitution of co-products for traditional feedstuffs. Increased agricultural productivity has allowed the global supply of crops available for non-biofuel uses to continue to grow over the long term. The future use of agricultural crops for biofuel resulting in a small increase in livestock feed costs, which will be offset to some extent by the use of co-products as feed and by increases in crop yields over time. Feed co-product output is expected to grow more slowly in the coming years. However, a number of new and emerging technologies may change the composition and further improve the nutritional quality and utility of feed co-products. New technologies and practices promise to change the complexion of the ethanol co-products market in the years ahead.

Co-products are supposed to be credited with the area of cropland required to produce the amount of feed they substitute. If co-products are taken into account, the net use of feedstocks decline. By adding co-products substituted for grains and oilseeds the land required for cultivation of feedstocks declines from about 2% to 1.5% net land requirement of the global crop area. Moreover, it is important to include the co-products in GHG assessment, because of their potential impact on the overall emissions. Most existing biofuel regulations significantly undervalue the contribution of co-products when assessing the net land use and GHG impacts of biofuel production. In the future accurate co-products accounting is of increasing importance.

Growth in the use of agricultural commodities for biofuels is expected to continue through to 2020, but growth rates will slow in key producing countries as government-imposed limits on grain use for biofuels are reached and advanced biofuels capacity is expected to expand only slowly. The second reason for moderation in the growth in the use of agricultural commodities for biofuels is the expectation that future growth in biofuels production will primarily come from new feedstocks that currently have no or limited application in the animal feed market, such as perennial grasses (*Miscanthus* sp. and *A.donax*), agricultural residues, algae and other materials.

## Figures and Tables

**Table 1:** Statistical estimates of minimum and maximum values of global bioenergy potential (EJ/year).

Studies Referring to 2050	Low Range	High Range
Smeets *et al.* [15]	215	1272
IEA Bioenergy [16]	50	1500
Dornburg *et al.* [17]	200	500
IPPC [18]	50	500
Haberl *et al.* [19]	160	270
Global Energy Assessment [20]	80	140

**Table 2:** Chemical composition of *A. donax*.

Hemi-Cellulose (%)	Cellulose (%)	Lignin (%)	Ashes (%)	References
24.2	41.6	24.9	3.2	[52]
34.8	20.9	23.0	n.d.	[53]
25.61 ± 0.07	33.85 ± 0.06	24.02 ± 0.04	5.04 ± 0.03	[54]
24.4 ± 0.52	39.1 ± 0.25	19.2 ± 3.25	4.2 ± 0.67	[55]
14.5	39.6	24.3	5.3	[56]
35.27 ± 2.80	31.10 ± 1.03	18.49 ± 0.10	n.d.	[57]

Source: Respective authors’ data.

## Abbreviations

The following abbreviations are used in this manuscript:

CO_2_: carbon dioxide
DDG: dried distillers’ grains
DDGS: dried distillers’ grains with solubles
dLUC: direct land use change
FAO: Food and Agriculture Organization of the United Nations
DME: dimethyl ether
GHG: greenhouse gas
ILUC: indirect land use change
OECD: Organisation for Economic Cooperation and Development
RED: renewable energy directive
RFS: renewable fuel standard
WDG: Wet distillers’ grain
CGF: corn gluten feed
