# Correction: Popp, J., *et al*. Biofuels and Their Co-Products as Livestock Feed: Global Economic and Environmental Implications. *Molecules* 2016, *21*, 285.

**DOI:** 10.3390/molecules21040546

**Published:** 2016-04-23

**Authors:** József Popp, Mónika Harangi-Rákos, Zoltán Gabnai, Péter Balogh, Gabriella Antal, Attila Bai

**Affiliations:** 1 Institute of Sectoral Economics and Methodology, Faculty of Economics and Business, University of Debrecen, Debrecen 4032, Hungary; popp.jozsef@econ.unideb.hu (J.P.); rakos.monika@econ.unideb.hu (M.H.-R.); balogh.peter@econ.unideb.hu (P.B.); 2 Institute of Business Economics, Faculty of Economics and Business, University of Debrecen, Debrecen 4032, Hungary; zoltangabnai@gmail.com (Z.G.); bai.attila@econ.unideb.hu (A.B.)

The authors wish to make the following correction to their paper [1]. In Figure 3, the measurement units are wrong, and should be billion Litre—bl L, not million Litre—mln L. The correct version of Figure 3 is as follows:

The authors would like to apologize for any inconvenience caused to the readers by this change.

## Figures and Tables

**Figure 3:**
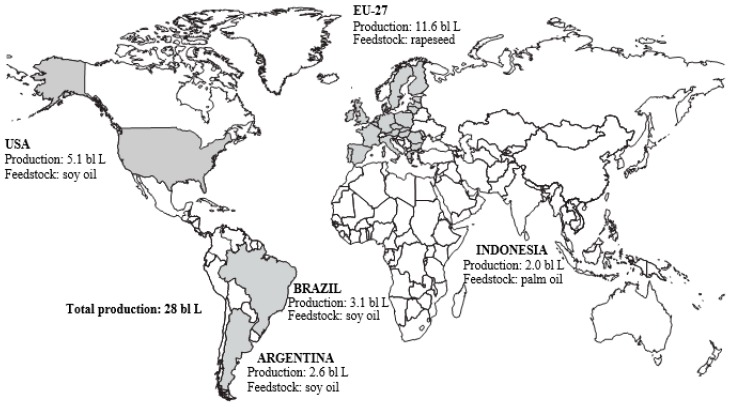
Word biodiesel production, average 2012–2014 [5].
